# Prognostic Value of MicroRNA-20b in Acute Myeloid Leukemia

**DOI:** 10.3389/fonc.2020.553344

**Published:** 2021-02-18

**Authors:** Zhiheng Cheng, Yifeng Dai, Wenhui Huang, Qingfu Zhong, Pei Zhu, Wenjuan Zhang, Zhihua Wu, Qing Lin, Huoyan Zhu, Longzhen Cui, Tingting Qian, Cong Deng, Lin Fu, Yan Liu, Tiansheng Zeng

**Affiliations:** ^1^ Department of Hematology, The Second Affiliated Hospital of Guangzhou Medical University, Guangzhou, China; ^2^ Department of Pathology and Medical Biology, University of Groningen, University Medical Center Groningen, Groningen, Netherlands; ^3^ Translational Medicine Center, State Key Laboratory of Respiratory Disease, The Second Affiliated Hospital of Guangzhou Medical University, Guangzhou, China; ^4^ Translational Medicine Center, Huaihe Hospital of Henan University, Kaifeng, China; ^5^ Department of Clinical laboratory, The Second Affiliated Hospital of Guangzhou Medical University, Guangzhou, China; ^6^ Department of Hematology, Huaihe Hospital of Henan University, Kaifeng, China; ^7^ Guangdong Provincial Education Department Key Laboratory of Nano-Immunoregulation Tumor Microenvironment, The Second Affiliated Hospital, Guangzhou Medical University, Guangzhou, China

**Keywords:** acute myeloid leukemia, miR-20b, allogeneic hematopoietic stem cell transplantation, chemotherapy, prognosis

## Abstract

Acute myeloid leukemia (AML) is a highly heterogeneous disease that requires fine-grained risk stratification for the best prognosis of patients. As a class of small non-coding RNAs with important biological functions, microRNAs play a crucial role in the pathogenesis of AML. To assess the prognostic impact of miR-20b on AML in the presence of other clinical and molecular factors, we screened 90 AML patients receiving chemotherapy only and 74 also undergoing allogeneic hematopoietic stem cell transplantation (allo-HSCT) from the Cancer Genome Atlas (TCGA) database. In the chemotherapy-only group, high miR-20b expression subgroup had shorter event-free survival (EFS) and overall survival (OS, both *P* < 0.001); whereas, there were no significant differences in EFS and OS between high and low expression subgroups in the allo-HSCT group. Then we divided all patients into high and low expression groups based on median miR-20b expression level. In the high expression group, patients treated with allo-HSCT had longer EFS and OS than those with chemotherapy alone (both *P* < 0.01); however, there were no significant differences in EFS and OS between different treatment subgroups in the low expression group. Further analysis showed that miR-20b was negatively correlated with genes in “ribosome,” “myeloid leukocyte mediated immunity,” and “DNA replication” signaling pathways. *ORAI2*, the gene with the strongest correlation with miR-20b, also had significant prognostic value in patients undergoing chemotherapy but not in the allo-HSCT group. In conclusion, our findings suggest that high miR-20b expression is a poor prognostic indicator for AML, but allo-HSCT may override its prognostic impact.

## Introduction

Acute myeloid leukemia (AML) is a clinically and genetically heterogeneous disease characterized by dysplasia and dysdifferentiation of primitive and immature myeloid cells in bone marrow and peripheral blood (1). The treatment and prognosis of AML patients are still very heterogeneous, although the French-American-British (FAB) classification based on cell morphology and immunology and the World Health Organization (WHO) classification based on cytogenetics have continuously refined AML. The development of molecular genetics also provides new prognostic markers for AML, such as *NPM1* mutation without *FLT3-ITD* and double *CEBPA* mutations are proved to be good prognostic indicators, while *FLT3-ITD*, *ASXL1*, *RUNX1* and *TP53* mutations are poor prognostic factors (2). In addition, altered gene expression is associated with the prognosis and may guide the treatment of AML patients. We found that AML patients with high expression of *FHL2*, *DDIT4*, *GOT1* and *iASPP* tend to have shorter survival, while those with high expression of *PAK2* and *DOK7* have better outcomes (3–7). The role of epigenetics, which does not involve changes in DNA sequence, in leukemogenesis is of increasing concern.

MicroRNAs (miRNAs) is a kind of small noncoding RNAs (about 20–24 nucleotides), which play an important role in post transcriptional gene regulation through inhibiting target messenger RNAs (mRNAs) (8). From exploring the expression patterns of miRNAs in specific AML to gradually clarifying miRNAs as oncomiRNAs or tumor suppressors in many AML subtypes, the roles of miRNAs have become increasingly clear in leukemic processes, including differentiation, proliferation, self-renewal, survival, epigenetic regulation, and chemotherapy resistance (9–11). MiRNAs affect the development and progression of leukemia by directly targeting the mRNAs of known oncogenes or tumor suppressors or by cooperating with these proteins. Especially, miRNA profiles have proved to be a useful complement to AML prognostication. For instance, overexpression of miR-98 is associated with better outcome in AML patients receiving chemotherapy (12); high miR-99a expression and low miR-29 and miR-212 expression are reported as adverse prognostic indicators in AML (13–15). Moreover, it has been demonstrated that some pharmacologic agents can modulate specific miRNAs to achieve antileukemic effects (8), suggesting that miRNA expression signature may help to guide the complex treatment selection of AML.

MiR-20b belongs to the miR-106a-363 cluster, which forms a large and highly similar miRNA family with miR-106b-25 and miR-17-92 clusters, known as the miR-17 family (16). MiR-20b is upregulated and affects disease progression in many kinds of human cancers, such as breast cancer, gastric cancer, prostate cancer and non−small cell lung cancer. For instance, miR-20b can negatively regulate autophagy by targeting *RB1CC1*/*FIP200* in breast cancer cells (17). Another study showed that the survival is significantly poorer in gastric cancer patients with high miR-20b expression than those without (18). Guo J et al. found that miR-20b promotes cellular proliferation and migration through directly regulating *PTEN* in prostate cancer (19). However, the indicative value of miR-20b in the prognosis and treatment of AML remains unclear. As an effective treatment, allogeneic hematopoietic stem cell transplantation (allo-HSCT) can reduce the recurrence of AML and promote survival by significantly reducing the residual disease of leukemia (20). Here, we analyzed the prognostic value and possible mechanism of miR-20b in AML, and delineated the treatment effect of allo-HSCT in AML patients with high miR-20b expression.

## Materials and Methods

### Patients

We collected 164 AML patients with miR-20b expression data and complete clinical information from the Cancer Genome Atlas (TCGA) database (https://tcga-data.nci.nih.gov/tcga) (21), of whom 90 patients received chemotherapy only and another 74 also received allo-HSCT. The main chemotherapy regimen for all patients is 7 + 3 (“7” refers to Cytarabine given daily for 7 days, “3” refers to Idarubicin given daily for 3 days). We obtained miR-20b expression data in peripheral blood of all patients at diagnosis. Clinical information at diagnosis, including age, sex, white blood cell (WBC) count, percentages of bone marrow (BM) and peripheral blood (PB) blasts, French-American-British (FAB) subtype, karyotype, risk stratification, and recurrent gene mutations, were described and analyzed. Overall survival (OS) and event-free survival (EFS) were the goal endpoints of this study. OS is defined as the time from diagnosis to death or loss of follow-up. EFS is defined as the time from diagnosis to removal from the study due to no complete remission, relapse, death or loss of follow-up.

### Statistical Analysis

Descriptive statistical methods were used to summarize the clinical and molecular characteristics of the patients. Continuous data were expressed by median with range, and categorical data were represented by frequency with percentage. Mann-Whitney *U* test and chi-square test were used for the comparison of numerical data and categorical data, respectively. Univariate (Kaplan-Meier method and log-rank test) and multivariate (Cox proportional risk model) analyses were used to assess whether miR-20b expression could predict EFS and OS. Spearman correlation analysis was used to screen gene expression profiles related to miR-20b. Then we used miRcode (http://www.mircode.org/index.php) to predict the target genes of miR-20b and intersected them with the related genes. Finally, we used Metascape (http://metascape.org/) to perform pathway enrichment analysis of intersection genes. We also did a survival analysis on the gene with the strongest correlation with miR-20b, and used another independent cohort (GSE12417) for further verification. A two-tailed *P* < 0.05 was defined as statistically significant. The SPSS 25.0 statistical software and R 3.5.0 software were used for statistical analyses, and the graphics were drawn by GraphPad Prism 8.0 software.

## Results

### Comparison of Clinical and Molecular Characteristics of High and Low miR-20b Expression Subgroups in Different Treatment Groups

All patients were divided into the chemotherapy-only group and the allo-HSCT group based on treatment modalities. Each group was further divided into high and low expression subgroups according to their respective median miR-20b expression levels. Comparison of clinical and molecular characteristics between the high and low expression subgroups in different treatment groups is shown in **Table 1**.

**Table 1:** Clinical and molecular characteristics of patients in different treatment groups.

Characteristics	Chemotherapy-only group				Allo-HSCT group		
	High miR-20b (n = 45)	Low miR-20b (n = 45)	*P*		High miR-20b (n = 37)	Low miR-20b (n = 37)	*P*
Age/years, median (range)	68 (33-88)	62 (22-82)	0.006^*^		53 (18-65)	49 (21-72)	0.387^*^
Age group/n (%)			0.013^§^				0.601^§^
<60 years	9 (20.0)	20 (44.4)			26 (70.3)	28 (75.7)	
≥60 years	36 (80.0)	25 (55.6)			11 (29.7)	9 (24.3)	
Gender/n (%)			0.396^§^				0.348^§^
Male	27 (60.0)	23 (51.1)			23 (62.2)	19 (51.4)	
Female	18 (40.0)	22 (48.9)			14 (37.8)	18 (48.6)	
WBC/×10^9^/L, median (range)	9.9 (0.7-297.4)	34 (1.4-298.4)	0.090^*^		12 (0.6-88.1)	34 (1.2-223.8)	0.001^*^
BM blast/%, median (range)	71 (30-99)	72 (32-98)	0.569^*^		69 (30-95)	71 (34-100)	0.475^*^
PB blast/%, median (range)	16 (0-98)	51 (0-97)	0.043^*^		35 (0-90)	58.5 (8-96)	0.003^*^
FAB classification/n (%)							
M0	7 (15.6)	1 (2.2)	0.058^§^		7 (18.9)	2 (5.4)	0.152^§^
M1	9 (20.0)	11 (24.4)	0.612^§^		10 (27.0)	13 (35.1)	0.451^§^
M2	9 (20.0)	12 (26.7)	0.455^§^		8 (21.6)	11 (29.7)	0.425^§^
M3	0 (0.0)	0 (0.0)	1.000^§^		2 (5.4)	0 (0.0)	0.493^§^
M4	11 (24.4)	13 (28.9)	0.634^§^		5 (13.5)	9 (24.3)	0.235^§^
M5	7 (15.6)	6 (13.3)	0.764^§^		3 (8.1)	1 (2.7)	0.615^§^
M6	0 (0.0)	2 (4.4)	0.494^§^		1 (2.7)	0 (0.0)	1.000^§^
M7	2 (4.4)	0 (0.0)	0.494^§^		1 (2.7)	1 (2.7)	1.000^§^
Cytogenetics (WHO)/n (%)							
Normal	22 (48.9)	22 (48.9)	1.000^§^		16 (43.2)	18 (48.6)	0.641^§^
Complex karyotype	10 (22.2)	2 (4.4)	0.013^§^		11 (29.7)	1 (2.7)	0.002^§^
8 Trisomy	0 (0.0)	0 (0.0)	1.000^§^		1 (2.7)	5 (13.5)	0.199^§^
inv(16)/CBFβ-MYH11	0 (0.0)	7 (15.6)	0.012^§^		0 (0.0)	5 (13.5)	0.054^§^
11q23/MLL	2 (4.4)	1 (2.2)	1.000^§^		2 (5.4)	1 (2.7)	1.000^§^
-7/7q-	3 (6.7)	0 (0.0)	0.242^§^		2 (5.4)	1 (2.7)	1.000^§^
t(15;17)/PML-RARA	0 (0.0)	0 (0.0)	1.000^§^		2 (5.4)	0 (0.0)	0.494^§^
t(9;22)/BCR-ABL1	0 (0.0)	1 (2.2)	1.000^§^		1 (2.7)	1 (2.7)	1.000^§^
t(8;21)/RUNX1-RUNX1T1	0 (0.0)	6 (13.3)	0.026^§^		0 (0.0)	1 (2.7)	1.000^§^
Others	8 (17.8)	6 (13.3)	0.561^§^		2 (5.4)	4 (10.8)	0.674^§^
Risk/n (%)							
Good	0 (0.0)	13 (28.9)	0.000^§^		2 (5.4)	6 (16.7)	0.152^§^
Intermediate	26 (60.5)	24 (53.3)	0.500^§^		18 (48.6)	23 (63.9)	0.190^§^
Poor	17 (39.5)	8 (17.8)	0.024^§^		17 (45.9)	7 (19.4)	0.016^§^
Chemotherapy regimen/n (%)			0.009^§^				1.000^§^
7+3	22 (48.9)	34 (75.6)			35 (94.6)	36 (97.3)	
Others	23 (51.1)	11 (24.4)			2 (5.4)	1 (2.7)	
*FLT3-ITD*/n (%)			1.000^§^				0.167^§^
Presence	8 (17.8)	8 (17.8)			6 (16.2)	11 (29.7)	
Absence	37 (82.2)	37 (82.2)			31 (83.8)	26 (70.3)	
*NPM1*/n (%)			0.114^§^				0.295^§^
Mutation	11 (24.4)	18 (40.0)			8 (21.6)	12 (32.4)	
Wild type	34 (75.6)	27 (60.0)			29 (78.4)	25 (67.6)	
*CEBPA*/n (%)			1.000^§^				0.005^§^
Single mutation	1 (2.2)	2 (4.4)			0 (0.0)	5 (13.5)	
Double mutation	0 (0.0)	0 (0.0)			0 (0.0)	3 (8.1)	
Wild type	44 (97.8)	43 (95.6)			37 (100)	29 (78.4)	
*DNMT3A*/n (%)			0.814^§^				0.588^§^
Mutation	13 (28.9)	12 (26.7)			10 (27.0)	8 (21.6)	
Wild type	32 (71.1)	33 (73.3)			27 (73.0)	29 (78.4)	
*IDH1*/*IDH2*/n (%)			0.581^§^				1.000^§^
Mutation	7 (15.6)	9 (20.0)			9 (24.3)	9 (24.3)	
Wild type	38 (84.4)	36 (80.0)			28 (75.7)	28 (75.7)	
*RUNX1*/n (%)			0.714^§^				0.261^§^
Mutation	5 (11.1)	3 (6.7)			6 (16.2)	2 (5.4)	
Wild type	40 (88.9)	42 (93.3)			31 (83.8)	35 (94.6)	
*MLL-PTD*/n (%)			1.000^§^				0.615^§^
Presence	2 (4.4)	3 (6.7)			3 (8.1)	1 (2.7)	
Absence	43 (95.6)	42 (93.3)			34 (91.9)	36 (97.3)	
*NRAS/KRAS*/n (%)			0.764^§^				1.000^§^
Mutation	6 (13.3)	7 (15.6)			4 (10.8)	3 (8.1)	
Wild type	39 (86.7)	38 (84.4)			33 (89.2)	34 (91.9)	
*TET2*/n (%)			0.215^§^				0.615^§^
Mutation	8 (17.8)	4 (8.9)			1 (2.7)	3 (8.1)	
Wild type	37 (82.2)	41 (91.1)			36 (97.3)	34 (91.9)	
*TP53*/n (%)			0.004^§^				0.115^§^
Mutation	10 (22.2)	1 (2.2)			4 (10.8)	0 (0.0)	
Wild type	35 (77.8)	44 (97.8)			33 (89.2)	37 (100)	
Relapse/n (%)			1.000^§^				0.619^§^
Yes	16 (35.6)	16 (35.6)			24 (64.9)	26 (70.3)	
No	29 (64.4)	29 (64.4)			13 (35.1)	11 (29.7)	

WBC, white blood cell; BM, bone marrow; PB, peripheral blood; FAB, French American British; 7 + 3, “7” refers to Cytarabine given daily for 7 days, “3” refers to Idarubicin given daily for 3 days.

‘*’denotes Mann-Whitney U test; ‘§’ denotes chi-square test.

In the chemotherapy-only group, high miR-20b expression subgroup had more old patients (≥60 years, *P* = 0.013), lower percentage of PB blasts (*P* = 0.043), more patients with complex karyotype (*P* = 0.013), fewer patients with *RUNX1-RUNX1T1* (*P* = 0.026) and *CBFβ-MYH11* (*P* = 0.012), more poor-risk patients (*P* = 0.024), fewer good-risk patients (*P* < 0.001), and more frequent *TP53* mutation (*P* = 0.004) than low expression subgroup. A higher proportion of “7+3” chemotherapy regimen in the low expression subgroup (*P* = 0.009). There were no significant differences in gender ratio, WBC count, BM blasts, FAB and other WHO classification, relapse rate, and frequency of other common gene mutations (*FLT3-ITD*, *CEBPA*, *DNMT3A*, *NPM1*, *RUNX1*, *IDH1*/*IDH2*, *NRAS*/*KRAS*, *TET2* and *MLL-PTD*) between the two subgroups.

In the allo-HSCT group, high miR-20b expression subgroup had lower WBC count (*P* = 0.001) and percentage of PB blasts (*P* = 0.003), more patients with complex karyotype (*P* = 0.002), more poor-risk patients (*P* = 0.016), and less frequent *CEBPA* mutation (*P* = 0.005) than low expression subgroup. There were no significant differences in age, gender ratio, BM blasts, FAB and other WHO classification, chemotherapy regimen before transplantation (mainly “7+3”), relapse rate, and frequency of other common gene mutations (*FLT3-ITD*, *TP53*, *NPM1*, *DNMT3A*, *RUNX1*, *IDH1*/*IDH2*, *NRAS*/*KRAS*, *TET2* and *MLL-PTD*) between the two subgroups.

### Prognostic Significance of miR-20b in AML

**Figure 1:**
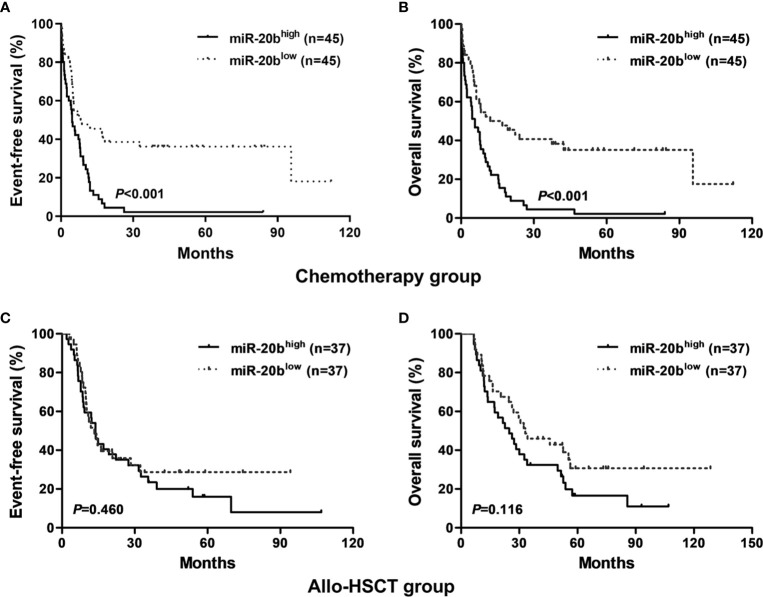
Kaplan-Meier curves of event-free survival (EFS) and overall survival (OS) in the chemotherapy-only and allo-HSCT groups. **(A**, **B)** In the chemotherapy-only group, high miR-20b expressers had shorter EFS and OS than low expressers. **(C**, **D)** In the allo-HSCT group, EFS and OS were not significantly different between high and low miR-20b expressers.

We found that patients with high miR-20b expression had shorter EFS and OS than those with low expression in the chemotherapy-only group (both *P* < 0.001, **Figures 1A, B**); whereas, there were no significant differences in EFS and OS between high and low expression subgroups in the allo-HSCT group (**Figures 1C, D**).

**Figure 2:**
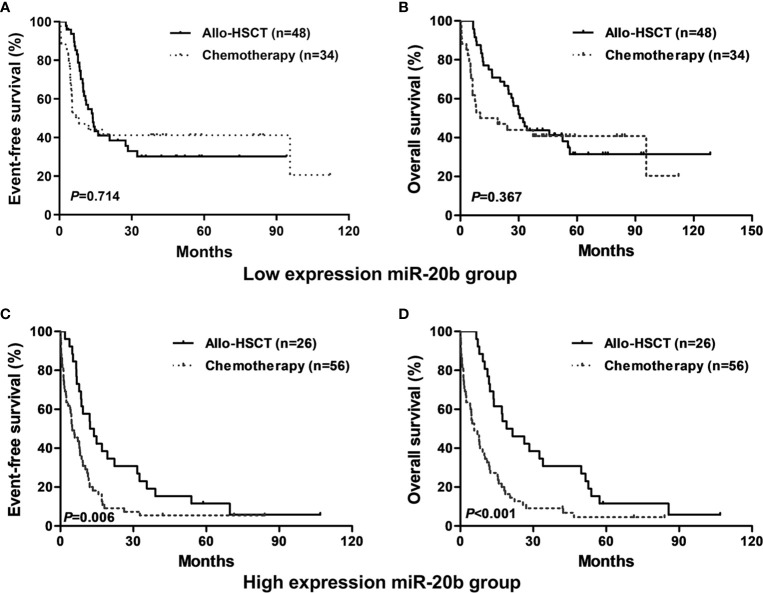
Kaplan-Meier curves of event-free survival (EFS) and overall survival (OS) in high and low miR-20b expression groups. **(A**, **B)** In low miR-20b expressers, EFS and OS were not significantly different between patients treated with chemotherapy-only and allo-HSCT. **(C**, **D)** In high miR-20b expressers, patients treated with allo-HSCT had longer EFS and OS than those underwent chemotherapy-only.

In the entire cohort, patients treated with allo-HSCT had longer EFS and OS than those with chemotherapy alone in the high expression group (both *P* < 0.01, **Figures 2C, D**); however, there were no significant differences in EFS and OS between different treatment subgroups in the low expression group (**Figures 2A, B**).

### Univariate and Multivariate Analyses in Different Treatment Groups

To evaluate the prognostic value of miR-20b expression in the presence of other clinical and molecular factors, we included the following dichotomous variables in univariate and multivariate Cox regression analyses: miR-20b expression levels (high vs. low), WBC count (≥20 vs. <20 × 10^9^/L), *FLT3-ITD* (positive vs. negative), and three common gene mutations (*NPM1*, *DNMT3A* and *TP53*; mutated vs. wild).

In the chemotherapy-only group (**Table 2**), univariate Cox regression analysis showed that high miR-20b expression had adverse effect on EFS and OS (both *P* < 0.001), and that *TP53* mutation contributed to poor OS and EFS (both *P* < 0.01); multivariate Cox regression analysis indicated that high miR-20b expression and *TP53* mutation were independent risk factors for OS and EFS even in the presence of other covariates (all *P* < 0.05).

**Table 2:** Univariate and multivariate analyses of EFS and OS in the chemotherapy-only group.

Variables	EFS			OS	
	HR (95%CI)	*P*		HR (95%CI)	*P*
Univariate analysis					
MiR-20b (high vs. low)	2.417 (1.484–3.934)	0.000		2.479 (1.528–4.022)	0.000
WBC (≥20 vs. <20 × 10^9^/L)	0.964 (0.608–1.528)	0.876		0.936 (0.591–1.484)	0.779
*FLT3-ITD* (positive vs. negative)	1.261 (0.703–2.260)	0.436		1.192 (0.665–2.136)	0.555
*NPM1* (mutated vs. wild)	1.120 (0.687–1.827)	0.649		1.044 (0.640–1.704)	0.862
*DNMT3A* (mutated vs. wild)	1.407 (0.852–2.322)	0.182		1.432 (0.868–2.362)	0.160
*TP53* (mutated vs. wild)	2.949 (1.510–5.761)	0.002		2.898 (1.487–5.649)	0.002
Multivariate analysis					
MiR-20b (high vs. low)	2.346 (1.355–4.061)	0.002		2.407 (1.391–4.166)	0.002
WBC (≥20 vs. <20 × 10^9^/L)	1.293 (0.769–2.175)	0.333		1.320 (0.783–2.225)	0.297
*FLT3-ITD* (positive vs. negative)	1.182 (0.626–2.231)	0.606		1.143 (0.607–2.152)	0.678
*NPM1* (mutated vs. wild)	1.316 (0.718–2.411)	0.374		1.204 (0.661–2.192)	0.544
*DNMT3A* (mutated vs. wild)	1.202 (0.677–2.134)	0.529		1.242 (0.709–2.177)	0.448
*TP53* (mutated vs. wild)	2.585 (1.213–5.510)	0.014		2.409 (1.138–5.096)	0.022

EFS, Event-free survival; OS, Overall survival; HR, hazard ratio; CI, confidence interval; WBC, white blood cell.

In the allo-HSCT group (**Table 3**), univariate Cox regression analysis indicated that *TP53* mutation was associated with shorter OS (*P* = 0.013); multivariate Cox regression analysis showed that *TP53* mutation was an independent risk factor for OS (*P* = 0.011), and that *FLT3-ITD* was an independent risk fact for EFS (*P* = 0.044), whereas, miR-20b expression had no independent effect on EFS and OS.

**Table 3:** Univariate and multivariate analyses of EFS and OS in the allo-HSCT group.

Variables	EFS			OS	
	HR (95%CI)	*P*		HR (95%CI)	*P*
Univariate analysis					
MiR-20b (high vs. low)	1.222 (0.716–2.085)	0.462		1.530 (0.897–2.611)	0.119
WBC (≥20 vs. <20 × 10^9^/L)	1.327 (0.778–2.261)	0.299		1.015 (0.595–1.731)	0.956
*FLT3-ITD* (positive vs. negative)	1.719 (0.915–3.229)	0.092		1.588 (0.848–2.972)	0.148
*NPM1* (mutated vs. wild)	0.907 (0.494–1.666)	0.753		0.910 (0.495–1.671)	0.761
*DNMT3A* (mutated vs. wild)	1.184 (0.643–2.178)	0.588		1.320 (0.715–2.438)	0.374
*TP53* (mutated vs. wild)	1.828 (0.651–5.133)	0.252		3.920 (1.334–11.520)	0.013
Multivariate analysis					
MiR-20b (high vs. low)	1.330 (0.739–2.395)	0.341		1.422 (0.778–2.597)	0.252
WBC (≥20 vs. <20 × 10^9^/L)	1.619 (0.887–2.955)	0.117		1.311 (0.711–2.417)	0.386
*FLT3-ITD* (positive vs. negative)	2.062 (1.021–4.163)	0.044		1.899 (0.945–3.819)	0.072
*NPM1* (mutated vs. wild)	0.670 (0.333–1.348)	0.262		0.752 (0.374–1.514)	0.425
*DNMT3A* (mutated vs. wild)	1.219 (0.642–2.313)	0.544		1.419 (0.741–2.717)	0.291
*TP53* (mutated vs. wild)	2.305 (0.746–7.118)	0.147		4.596 (1.414–14.937)	0.011

EFS, Event-free survival; OS, Overall survival; HR, hazard ratio; CI, confidence interval; WBC, white blood cell.

### Potential Mechanism of miR-20b in AML

**Figure 3:**
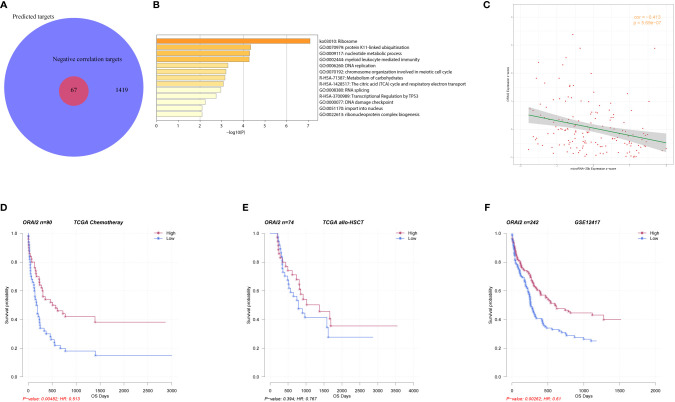
Biologic insight into miR-20b in AML. **(A**, **B)** Genes related to miR-20b and their pathway enrichment analysis. **(C)** Correlation analysis of miR-20b and ORAI2. **(D**–**F)** Survival analysis of ORAI2 in different groups.

To further explore the mechanism of miR-20b in AML, we first predicted that 1419 genes were potential target genes of miR-20b, and 67 of them had significant negative correlations with miR-20b expression based on Spearman correlation analysis (**Figure 3A**, details of 67 genes are in **Table S1**). Pathway enrichment analysis showed that 67 genes related to miR-20b were mainly involved in “ribosome,” “protein K11-linked ubiquitination,” “nucleotide metabolic process,” “myeloid leukocyte mediated immunity,” “DNA replication,” “RNA splicing,” “transcriptional regulation by TP53” signaling pathways (**Figure 3B**). Among these 67 genes, *ORAI2* (ORAI calcium release-activated calcium modulator 2) had the strongest correlation with miR-20b (*r*=-0.413, *P* < 0.001, **Figure 3C**). Survival analysis showed that patients with high *ORAI2* expression had longer OS than those with low expression in the chemotherapy group (*P* = 0.005, **Figure 3D**); whereas, no similar positive result in the allo-HSCT group (**Figure 3E**). In another validation dataset (GSE12417), high *ORAI2* expression was also accompanied by better survival (*P* = 0.003, **Figure 3F**).

## Discussion

Our study showed that high miR-20b expression is an adverse prognostic factor in AML patients receiving chemotherapy only. No adverse effect of miR-20b expression on survival was observed in patients who received allo-HSCT, and as demonstrated by the second analysis, it might be overcome with allo-HSCT. On the other hand, AML patients with low miR-20b expression could not obtain survival benefit from allo-HSCT in the study.

The mechanisms of miR-20b in various tumors have been widely studied. Danza K, et al. found that the down-regulation of miR-20b can lead to the increased expression of *HIF1A*, *MDR1* and *HIPK2*, which is related to the chemotherapeutic response in gastric cancer (22); Zhou W et al. found that miR-20b can directly bind to the 3’ untranslated region (UTR) of the phosphatase and tensin homolog (*PTEN*) mRNA and suppress its translation, thereby promoting growth and metastasis of breast cancer cells (23); and the mechanism by which miR-20b directly regulates *PTEN* to promote cell proliferation, migration and invasion has also been found in liver, prostate, and esophageal cancers (24–26). Overexpression of miR-20b-5p promotes the malignant behavior of breast cancer stem cells by bidirectional regulation of *CCND1* and *E2F1* (27); while the deletion of miR-20b and miR-17 enhances the resistance of breast cancer to taxol by inhibiting the expression of *NCOA3* (28). In addition, miR-20b inhibits the proliferation, migration and invasion of osteosarcoma cells and colon cancer cells by targeting *HIF-1α* and *cyclinD1*, respectively (29, 30). High miR-20b expression indicates poor prognosis in laryngeal squamous cell carcinoma and prostate cancer (25, 31). The expression of miR-20b-5p is also elevated in chronic lymphocytic leukemia (CML), but it is an indicator of favorable prognosis (32). We found that high miR-20b expression indicates poor prognosis in AML patients receiving chemotherapy only, and it coexists with known adverse prognostic factors such as *TP53* mutation and old age but not with known favorable prognostic factors such as *RUNX1-RUNX1T* and *CBFβ-MYH11*. These results support that miR-20b expression may contribute to some of the invasive features of AML.

We further explored the potential mechanism of miR-20b in AML and found that the expression of genes involved in “ribosome,” “nucleotide metabolic process,” “myeloid leukocyte mediated immunity,” and “DNA replication” signaling pathways is negatively correlated with miR-20b expression. Ribosome modulation participates in the exacerbation of T-cell acute lymphoblastic leukemia (T-ALL) caused by 6q deletion (33). Abnormal DNA replication in hematopoietic cells is also common to various blood diseases (34). We found that *ORAI2* has the strongest negative correlation with miR-20b. It is a tetraspanning plasma membrane protein that forms Ca^2+^ release-activated Ca^2+^ channels with ORAI1 and ORAI3 to mediate Ca^2+^ influx (35). ORAI2 regulates the migration and colonization of oral cancer cells by inhibiting Akt/mTOR/NF-κB signaling pathway (36). Diez-Bello R, et al. found that ORAI2 can regulate store-operated calcium entry to promote cell migration and FAK tyrosine phosphorylation in the AML cell line HL60 (37). MiR-20b may participate in the process of AML by targeting genes in the above signaling pathways, but the mechanism of miR-20b in leukemogenesis needs further study.

More and more gene mutations are incorporated into the prognosis and risk stratification and treatment outcome-prediction of AML. Allo-HSCT can improve the prognosis of CN-AML patients with *DNMT3A* mutation but cannot triumph over the adverse prognostic effect of *FLT3-ITD*, and *TP53* mutation is associated with increased relapse-risk after allo-HSCT (38–40). *FLT3-ITD* and *DNMT3A* double mutation denote poor prognosis in AML patients even after allo-HSCT (41). Negative *FLT3-ITD*, *NPM1* and biallelic *CEBPA* mutations confer better prognosis in AML patients who receiving post-remission allo-HSCT than consolidation chemotherapy alone (42). Our survival analysis found that high miR-20b expression and *TP53* mutation are independent risk factors in AML patients received chemotherapy only, but the other widely-used genetic characteristics, such as *FLT3-ITD* and *NPM1* and *DNMT3A* mutations, are not associated with EFS and OS. Thus, it may be useful to incorporate miR-20b into prognostication. In the allo-HSCT group, miR-20b expression is not correlated with survival, suggesting that its adverse prognostic effect in AML may be conquered by allo-HSCT. Throughout the cohort, patients with high miR-20b expression benefit more from allo-HSCT, but the treatment modalities of those with low mir-20b expression do not affect survival. Therefore, allo-HSCT may be a better choice for AML patients with high miR-20b expression, but it may not be necessary for patients with low miR-20b expression.

In conclusion, our results suggest that high miR-20b expression is a poor prognostic factor in AML and these patients may benefit from allo-HSCT. Thus, it is reasonable to envision it as a marker for risk stratification and guidance for treatments in AML. Our study is limited by the small sample size and needs to be verified by a larger prospective population. The mechanism of miR-20b in AML, especially its association with ORAI2, requires further experimental verification.

## Data Availability Statement

Publicly available datasets were analyzed in this study. This data can be found here: https://tcga-data.nci.nih.gov/tcga.

## Author Contributions

ZC and YD collected and analyzed the data. ZC drafted the manuscript. ZC and YD revised the manuscript. WH, QZ, PZ, WZ, ZW, QL, HZ, LC, TQ, and CD participated in the data collection and discussion in the revision. LF, YL, and TZ conceived and led the study. All authors contributed to the article and approved the submitted version.

## Funding

This work was supported by grant from Xinjiang Joint Fund of National Natural Science Foundation of China (U1903117).

## Conflict of Interest

The authors declare that the research was conducted in the absence of any commercial or financial relationships that could be construed as a potential conflict of interest.
